# Effects of *Pleurotus eryngii* (mushroom) powder and soluble polysaccharide addition on the rheological and microstructural properties of dough

**DOI:** 10.1002/fsn3.1054

**Published:** 2019-05-14

**Authors:** Yuanyang Nie, Penghui Zhang, Chujun Deng, Linshuang Xu, Mingjun Yu, Wei Yang, Renyong Zhao, Bo Li

**Affiliations:** ^1^ College of Food Science and Technology Henan University of Technology Zhengzhou China; ^2^ Postdoctoral Research Base Henan Institute of Science and Technology Xinxiang China; ^3^ School of Food Science Henan Institute of Science and Technology Xinxiang China

**Keywords:** dough, microstructure, mushroom, polysaccharide, rheology, viscoelasticity

## Abstract

Adding a certain proportion of *Pleurotus eryngii* can improve the nutritional value of wheat‐flour foods and enhance the utilization of this mushroom. In this research, partial wheat flour was substituted with *P. eryngii* powder (PEP) or soluble polysaccharide (SPPE) at different addition levels, and the effects of PEP and SPPE on the rheological and microstructural properties of dough were investigated. Farinographic assay results suggested that PEP significantly (*p* < 0.05) increased the water absorption of wheat flour but decreased the development time and stability of dough significantly (*p* < 0.05). Furthermore, it was capable of providing weaker extensographic characteristics and harder dough with the increasing of PEP addition levels. The dynamic oscillatory tests indicated that the PEP addition approximately increased the storage (G′) and loss (G″) moduli in the entire frequency range, while the tan δ roughly decreased with the increasing of PEP addition levels, which could be attributed to the low solubility and strong water‐trapping capacity of the dietary fiber in PEP. Due to the good water solubility and easy formation of hydrogen bonds, the addition of SPPE had inconsistent results with the PEP addition. The inner microstructure of dough showed that the continuity of gluten networks had been disrupted by PEP and SPPE addition and then resulted in a weaker extension and harder dough. This research could provide a foundation for the application of PEP in wheat‐flour foods, and PEP addition levels of 2.5%–5.0% are recommended.

## 1 INTRODUCTION

*Pleurotus eryngii*, also called the king oyster mushroom, is a precious commercial edible fungus with high output and consumption in China (Sun, Hu, & Li, 2017). As a popular type of edible mushroom, *P. eryngii* has an attractive taste and high nutritional value, as it contains many biologically active substances, such as polysaccharides, lipids, peptides, sterols, and dietary fiber (Chen et al., 2012; Liu et al., 2010). It has also been highly valued as a functional food for its good immunomodulatory, antioxidant, antitumor, anti‐inflammatory, antihyperglycemia, hepatoprotective, and hypolipidemic effects (Chen et al., 2012; Jeong, Jeong, Gu, Islam, & Song, 2010; Ren, Wang, Guo, Yuan, & Yang, 2016; Sun et al., 2017; Yuan, Zhao, Rakariyatham, et al., 2017; Zhang et al., 2018). In recent years, many factories have been set up for the large‐scale cultivation of *P. eryngii*. The annual output of *P. eryngii* in China has exceeded 1,000,000 tons, and it has become the second‐largest mushroom variety under factory cultivation. However, there are few deep‐processing technologies and products for *P. eryngii* at present in China, and the main consumption pattern is fresh eating. As a result, the inconsistency between production and marketing is becoming increasingly acute with the fast growth of *P. eryngii* output. Therefore, developing deep‐processing technologies and products is necessary for the healthy and sustainable development of the *P. eryngii* industry.

Wheat‐flour foods are consumed at high levels in China and around the world. In recent years, many exogenous materials, such as wheat bran, black tea, purple sweet potato powder, banana pulp and peel, seed powder, fiber, nonstarch polysaccharides, and soy protein, have been added into noodles, bread, and steamed bread to improve their nutritional and functional properties (Du et al., 2016; Fu, Chang, & Shiau, 2015; Gökşen & Ekiz, 2016; Li, Zhu, Guo, Brijs, & Zhou, 2014; Ramli, Alkarkhi, Shin, Min‐Tze, & Easa, 2009; Santiago et al., 2016; Sim, Aziah, & Cheng, 2015; Song, Zhu, Pei, Ai, & Chen, 2013; Wandee et al., 2015; Zhu, Sakulnak, & Wang, 2016). Dough modulation is the basis for preparing most wheat‐flour foods. Dough is an integrated network composed by wheat flour, water, and other ingredients (Peng, Li, Ding, & Yang, 2017), in which starch granules fill within a continuous matrix formed by gluten (Edwards, Dexter, & Scanlon, 2002). Gluten is the major protein source in wheat flour, which is extremely important to form the viscoelastic structure of dough (Mirsaeedghazi, Emam‐Djomeh, & Mousavi, 2008). Rheological properties are closely associated with the viscoelasticity and mixing tolerance of dough, which also provide important reference for the processing properties of flour (Xu, Hu, Liu, Dai, & Zhang, 2017). The rheological properties of dough not only determine the processing characteristics during the manufacture of dough‐based products but also affect the quality of final products (McCann, Le Gall, & Day, 2016). Exogenous additives, like proteins, polysaccharides, and fibers, can produce significant effects on the rheological behavior of doughs, which is likely due to the interactions between exogenous additives and wheat proteins, and further affect the integrity and stability of the gluten network (Mirsaeedghazi et al., 2008; Morris & Morris, 2012; Rubel, Pérez, Manrique, & Genovese, 2015).

Given its excellent nutritional value and bioactivity, adding *P. eryngii* into wheat‐flour foods can improve the nutritional properties of products. In view of the huge consumption of wheat‐flour foods, replacing part of the wheat flour with *P. eryngii* can also consume a large number of mushrooms and thereby resolve the inconsistency between the production and marketing of *P. eryngii*. *P. eryngii* contains polysaccharides, dietary fiber, protein, and other low‐molecular‐weight components, which would affect the rheological properties of dough and therefore influence the qualities of dough‐based products. Studies have proved that mushroom powder can impact the rheological and structural properties of wheat dough and therefore the textural characteristics of products (Lu, Brennan, Serventi, Mason, & Brennan, 2016; Yuan, Zhao, Yang, McClements, & Hu, 2017). However, little information is available for the properties of wheat dough supplemented with single chemical component in mushrooms. Thus, the objective of this work was to investigate the effects of *P. eryngii* powder (PEP) and soluble polysaccharide on the rheological properties and microstructure of dough, so as to provide the basis for the processing of dough‐based foods containing *P. eryngii*.

## 2 MATERIALS AND METHODS

### 2.1 Materials

Fresh *P. eryngii* was obtained from Xinxiang Kanghong Edible Mushrooms Co., Ltd. Commercial wheat flour with 12.0% moisture, 1.5% fat, and 9.0% protein was obtained from COFCO Co., Ltd. Salt was purchased from the local market. High temperature‐resistant alpha‐amylase solution (30 U/mg) and glycosylase solution (100 U/mg) were purchased from Shanghai Ruji Bio‐Technology Co., Ltd. Alkaline protease solution (100 U/mg) was purchased from Shanghai Lanji Bio‐Technology Co., Ltd.; 2‐(4‐Morpholino)ethanesulfonic acid (MES) and tris(hydroxymethyl)aminomethane (TRIS) were bought from Sigma‐Aldrich Chemical Co. All other chemicals used in experiments were of analytical grades.

### 2.2 Preparation of *Pleurotus eryngii* powder

After removal of the root, the sliced fresh *P. eryngii* were sun‐cured outside and dehydrated to about 70%. Semidried *P. eryngii* were then dried in a 75°C draft drying cabinet for 5–6 hr. The dried mushrooms were milled using a laboratory‐scale pulverizer (Beijing Zhongxingweiye Instrument Co., Ltd.) and then screened (>80 mesh).

### 2.3 Preparation of soluble polysaccharide of *Pleurotus eryngii*

PEP was extracted with petroleum ether at 80°C for 2 hr to remove lipids and pigments. The residue was then extracted with hot distilled water (ratio of water to material of 20 ml/g) at 90°C for 1 hr and then centrifuged at 3,000 *g* for 15 min to remove the precipitate. This process was repeated twice, and the two extraction solutions were merged. Crude polysaccharide extraction solution was precipitated with 3‐fold volume anhydrous ethanol (ethanol final concentration, 75%) and then kept at 4°C for 24 hr. After centrifugation at 3,000 *g* for 15 min, the precipitate was washed with anhydrous ethanol and acetone, respectively, and then lyophilized as crude soluble polysaccharide of *P. eryngii* (SPPE).

### 2.4 Chemical composition of PEP

Moisture, ash, protein, lipid, and reduced sugar contents of PEP were measured by the standard approved methods 44–15, 08–01, 46–11, 30–10, and 80–68 of AACC International (2000). The total carbohydrate content was calculated as a difference: total—moisture—ash—protein—lipid (%; Yuan, Zhao, Yang, et al., 2017). The contents of total dietary fiber (TDF), insoluble dietary fiber (IDF), and soluble dietary fiber (SDF) in PEP were assayed by the enzymatic–gravimetric method of AOAC 991.43 (AOAC Official Method 991.43 [17th ed.], 2000). The soluble polysaccharide content of PEP was determined using a phenol–sulfuric acid method (Dubois, Gilles, Hamilton, Rebers, & Smith, 1956). Mineral elements were assayed by inductively coupled plasma atomic emission spectrometry (ICP, Optima 2100DV, PE Co.). The amino acid composition of PEP was detected by HPLC with OPA/FMOC‐Cl precolumn derivatization (Zeng et al., 2013). Monosaccharide composition analysis of SPPE was conducted according to the method of Sheng et al. (2007).

### 2.5 Preparation of composite flours

Wheat flour was replaced with PEP at the levels of 2.5%, 5.0%, 7.5%, 10.0%, 12.5%, and 15.0%, respectively, or replaced with SPPE at the levels of 1%, 2%, 3%, 4%, 5%, and 6%, respectively. Composite flours were prepared by mixing wheat flour and PEP or wheat flour and SPPE thoroughly.

### 2.6 Rheological characterization of dough

#### 2.6.1 Farinographic assays

The farinographic assay was carried out according to the AACCI approved method 54‐21 (AACC, 2000) using a farinograph equipment (Brabender, Duisburg, Germany) with a mixing bowl for 300 g flour. The farinographic parameters measured were water absorption, development time, and stability time.

#### 2.6.2 Extensographic assays

Extensographic properties of dough were measured by the AACCI approved method 54–10 (AACC, 2000) using an extensograph equipment (Brabender). The extensographic parameters measured were area (energy), maximum resistance (Rm), extensibility (Ex), and ratio between resistance and extensibility.

#### 2.6.3 Dynamic rheological assays

Dough for dynamic rheological assays was kneaded for 5 min using a 50 g farinograph (Brabender), and water absorption was optimized to make the consistency at the end of mixing centered in the range of 480–520 B.U. The dough was then covered with a plastic film immediately to avoid water losses, and cylindrical pieces of 3 cm diameter and 2 mm height were obtained from dough. Dynamic oscillatory tests were carried out in a Haake MARS III controlled stress oscillatory rheometer (Haake) at 25 ± 0.1°C using a 1 mm gap plate–plate sensor system. Test parameters referenced the method of Correa, Añón, Pérez, and Ferrero (2010). Storage modulus (G′), loss modulus (G″), and tan δ (G″/G′) were obtained as a function of frequency.

### 2.7 Scanning electron microscope observation of dough

Microstructures of doughs prepared with different levels of PEP and SPPE were performed using a Quanta 200 environmental scanning electron microscope (SEM; FEI, Hitachi). Dough samples were cut into 5 mm height and 3 cm diameter cylindrical pieces, which were dried by vacuum freeze drying. The dried dough slices with natural fracture surfaces were fixed by conducting resin and coated with gold and then observed at a voltage of 20 kV and high vacuum condition.

### 2.8 Statistical analysis

All experiments were performed at least in duplicate. Statistical analysis was performed by SPSS version 17.0 software for Windows (SPSS Inc.). One‐way analysis of variance (ANOVA) was used to test the significant differences between means, and a post hoc test (Dunnett's T3) was used to perform multiple comparisons between means at a *p < *0.05 significance level. Correlations between rheological characterization and PEP/SPPE addition levels and viscoelastic properties were performed with the Spearman correlation coefficient test.

## 3 RESULTS AND DISCUSSION

### 3.1 The chemical composition of PEP

The moisture content of fresh *P. eryngii* was 90.26%. After drying, milling, and sieving, the mushroom powder (>80 mesh) was obtained. The chemical composition and mineral element contents of PEP are shown in Table 1. The results showed that the primary components of PEP were polysaccharides, dietary fiber, and protein. The potassium content of PEP was high, and the ratio of potassium to sodium was 143. A diet high in potassium and low in sodium is conducive to preventing hypertension (Oliver, Cohen, & Neel, 1975) and cardiovascular disease (Chang et al., 2006). The zinc content of PEP was high, which is helpful for human growth (Ploysangam, Falciglia, & Brehm, 1997).

**Table 1:** Chemical composition and mineral element contents of *Pleurotus eryngii* powder

Component	Content (%)	Element	Content (mg/g)
Moisture	4.62	K	24.26
Ash	4.90	Ca	0.11
Crude fat	5.13	Na	0.17
Protein	16.39	Mg	1.05
Total carbohydrate	68.96	Fe	0.03
Reduced sugar	0.52	Cu	nd
Water‐soluble polysaccharide	35.45	Zn	0.07
TDF	34.03	Mn	nd
IDF	32.72	Cr	nd
SDF	1.31	K/Na	142.71

Note

IDF, insoluble dietary fiber; SDF, soluble dietary fiber; TDF, total dietary fiber.

The composition of amino acids in PEP is shown in Table 2. PEP contained 16.59 g amino acids per 100 g proteins, and the E/T value (ratio of essential amino acids to total amino acids) was 39%.

**Table 2:** Composition of amino acids in *Pleurotus eryngii* powder

Amino acid	Content (g/100 g proteins)	Amino acid	Content (g/100 g proteins)
Thr	0.82	Ser	0.71
Val	1.57	His	0.50
Met	0.23	Gly	0.97
Phe	0.72	Arg	1.14
Ile	0.83	Ala	1.25
Leu	1.19	Tyr	0.44
Lys	1.11	Cys	0.06
Asp	1.83	Pro	0.29
Glu	2.95	Total	16.59

The monosaccharide composition of SPPE was glucose (86.30%), galactose (9.63%), mannose (3.56%), and fucose (0.50%). Polysaccharides are the major bioactive component in *P. eryngii*, and many studies have demonstrated that they have many biological activities, such as antioxidant (Sun et al., 2017), anti‐inflammatory (Yuan, Zhao, Rakariyatham, et al., 2017), immunomodulatory (Jeong et al., 2010), and antitumor (Ren et al., 2016) activities. The analytical results suggested that adding a moderate amount of PEP could improve the deficiencies of wheat‐flour foods in nutrients such as dietary fiber, lysine, and potassium.

### 3.2 Farinographic behavior of composite flours

**Figure 1:**
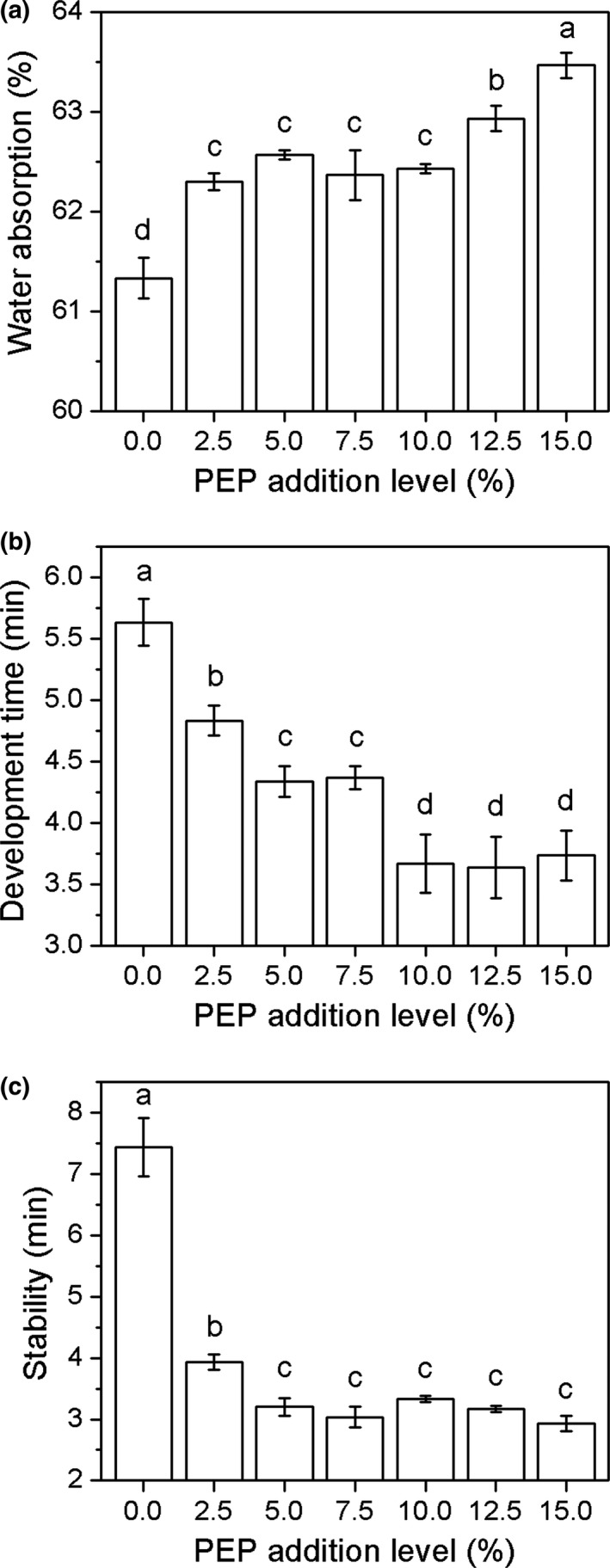
Farinographic properties of composite flour supplemented with PEP at various levels. (a) water absorption, (b) development time, and (c) stability time. Different lowercase letters on the error bars are significantly (*p* < 0.05) different

We observed that the water absorption of composite flour supplemented with 2.5% PEP increased significantly (*p < *0.05) compared to wheat flour, and the increase tended to be subtle until the 10% addition of PEP, finally reaching significance (*p < *0.05) at 12.5% and 15.0% addition levels (Figure 1a). As previously reported, the huge number of hydroxyl groups, such as polysaccharides and dietary fiber, caused the larger water absorption values (Goldstein, Ashrafi, & Seetharaman, 2010; Zhou et al., 2009). Correa et al. (2010) reported that modified cellulose addition increased the water absorption of wheat flour. Therefore, the increased water absorption of composite flour could be attributed to the large number of carbohydrates (68.96%) existed in PEP.

The development time and stability of dough for composite flour are shown in Figure 1b,c, respectively. The development time decreased to the minimum at a 10.0% PEP level, with almost no changes at the 12.5% and 15.0% levels. The farinograph stability significantly decreased (*p < *0.05) at a 2.5% PEP level and decreased nonsignificantly with further PEP addition. The stability value is closely related to flour strength, with a higher value representing stronger dough. Generally, the addition of exogenetic ingredients containing no gluten will weaken the gluten networks and lead to lower development time and stability of dough (Petitot, Boyer, Minier, & Micard, 2010).

### 3.3 Extensographic behavior of composite dough

**Figure 2:**
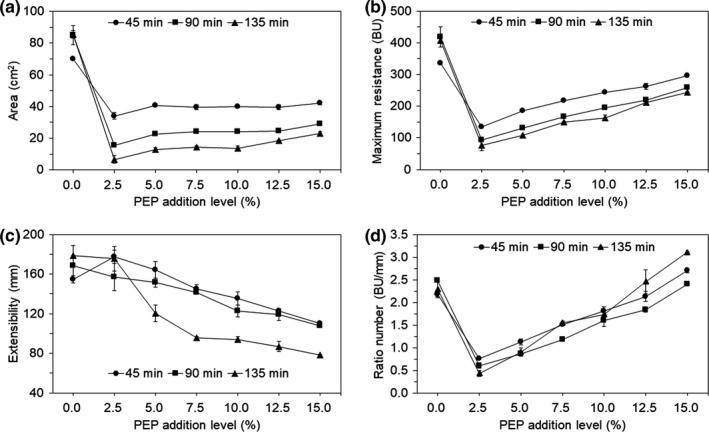
Extensographic properties of composite dough supplemented with PEP at various levels. Dough samples were rested for 45, 90, and 135 min, respectively. (a) area, (b) maximum resistance, (c) extensibility, and (d) ratio between resistance and extensibility

The extensographic properties of dough samples containing PEP are shown in Figure 2. After 45 min of fermentation, the area (energy) of the dough decreased significantly (*p < *0.05) at a 2.5% PEP level, increased more subtly at a 5.0% level, and increased nonsignificantly thereafter. The Ex of the dough with 2.5% and 5.0% PEP supplementation were both higher than that of the control group, and these properties decreased when the level of PEP was increased to 15.0%, which significantly (*ρ* = −0.978, *p < *0.01) negatively correlated with the PEP addition levels (Table 3). This indicated that a small amount (<5.0%) of PEP had a positive impact on the formation of the gluten network, while a larger amount (>5.0%) of PEP had a negative impact. This result was consistent with Ahmed, Almusallam, Al‐Salman, Abdulrahman, and Al‐Salem (2013), who reported that fiber addition decreased the area (energy) and Ex values of dough. For 90 and 135 min of fermentation, the variation trends of area (energy) were consistent with that of 45 min of fermentation. With PEP addition levels increasing, the Ex of the dough decreased. In addition, the composite dough with PEP had a lower Rm and ratio number than the control dough (with the exception of the ratio number at the 15.0% PEP level), with minimum values at a 2.5% PEP addition level. According to the research of Ribotta, Ausar, Beltramo, and León (2005), higher values of Rm and Ex indicate greater dough strength. This suggests that the addition of PEP weakened the extensographic properties of dough, overall. A possible reason is that partial wheat flour was substituted by PEP, which relatively decreased the wheat gluten content (dilution effect). It is well known that proteins in PEP cannot participate in the dough formation, which disrupts the well‐defined protein–starch complex in wheat‐flour dough, resulting in a weakening of dough (Sun, Zhang, Hu, Xing, & Zhuo, 2015). In addition, PEP contains a huge number of hydroxyl groups, such as polysaccharides and dietary fiber, which have strong water absorption and would compete for water with starch and proteins in wheat flour (water absorption effect). Furthermore, PEP could fill into the gluten network and thus affect the extensographic properties of the dough (filling effect).

**Table 3:** Rheological characterization of composite dough and their correlation with PEP/SPPE addition levels and viscoelastic properties

Rheological characterization	PEP/SPPE addition levels	G′ (PEP)	G″ (PEP)	tan δ (PEP)
Area (energy)	−0.474	0.055	0.023	−0.271
Rm	−0.037	0.487	0.459	−0.619
Ex	−0.978**	−0.875**	−0.898**	0.649*
Rm/Ex	0.516	0.879**	0.863**	−0.859**
G′ (PEP)	0.849*	1	0.997**	−0.880**
G″ (PEP)	0.866*	0.997**	1	−0.841*
tan δ (PEP)	−0.622	−0.880**	−0.841*	1
G′ (SPPE)	−0.051	0.471	0.443	−0.595
G″ (SPPE)	0.177	0.652	0.630	−0.721
tan δ (SPPE)	0.302	−0.235	−0.200	0.416

Note

Data are Spearman correlation coefficients. Significant correlations are shown as *p < 0.05 and **p < 0.01. Ex, extensibility; Rm, maximum resistance; Rm/Ex, ratio between maximum resistance and extensibility.

### 3.4 Viscoelastic properties of composite dough

**Figure 3:**
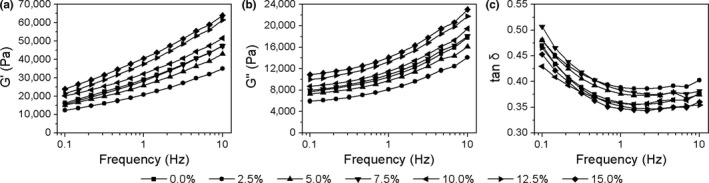
Typical frequency dependence of G′ (a), G″ (b), and tan δ (c) of composite dough supplemented with PEP at various levels

The viscoelastic properties of dough with PEP addition are presented in Figure 3. The results showed that G′ and G″ of all doughs increased with the increase of frequency (Figure 3a,b). All dough samples displayed higher G′ than G″ in the range of frequencies used in this study, indicating that all doughs exhibited more elastic behavior compared to viscous behavior with or without PEP addition (Shi, Wang, Li, & Adhikari, 2013). Meanwhile, G′ and G″ of dough first decreased at the 2.5% PEP level and then increased with the increase of the PEP addition level (Figure 3a,b), which both significantly (*ρ* = 0.849, *p < *0.05; *ρ* = 0.866, *p < *0.05) positively correlated with the PEP addition levels (Table 3). The tan δ values of dough at 2.5%, 5.0%, and 7.5% PEP levels were higher than those in the control group (Figure 3c), which revealed higher liquid‐like behavior instead of solid‐like behavior. This was probably caused by the dietary fiber in PEP, which could strengthen the water absorption ability of dough. As shown in the results of farinographic assays, the water absorption of composite flour supplemented with 2.5%, 5.0%, and 7.5% PEP increased significantly (*p < *0.05) compared to wheat flour, and then the increase tended to be nonsignificant until the 15% addition of PEP (Figure 1a). The significantly increased water promoted the swelling of wheat‐flour starch and proteins at the comparatively lower level (<2.5%) of PEP, which could increase the viscosity of the dough and make it softer. However, with increased PEP level (>7.5%), the tan δ of the dough roughly decreased (Figure 3c), indicating that the elasticity prevailed over the viscosity in the doughs with a larger amount (>7.5%) of PEP addition. This might be attributed to the low solubility and strong water‐trapping capacity of the dietary fiber in PEP. According to the research of Blanco Canalis, Steffolani, León, and Ribotta (2017), β‐glucans in oat fiber have a high proportion of β‐(1 → 4) linkages which could promote hydrogen bond formation between polymer chains and with water molecules as well. Furthermore, because of the low solubility, nonstarch polysaccharides cannot enter the aqueous phase of dough, which resulted in the water distribution and dough viscosity changed. Consequently, the increased PEP (>7.5%) imparted greater water‐holding characteristics than wheat‐flour starch and proteins, which ultimately resulted in an elastic and firmer, but less viscous, dough. The variational tendency in the G′ and G″ of PEP dough both significantly (*ρ* = −0.875, *p < *0.01; *ρ* = −0.898, *p < *0.01) negatively correlated with that in the extensographic properties of Ex, while tan δ significantly (*ρ* = 0.649, *p < *0.05) positively correlated with the Ex (Table 3).

**Figure 4:**
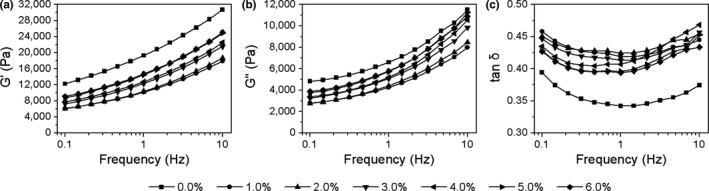
Typical frequency dependence of G′ (a), G″ (b), and tan δ (c) of composite dough supplemented with SPPE at various levels

Polysaccharides are one of the primary components and the most important bioactive substances of *P. eryngii*. Therefore, water‐soluble polysaccharides of *P. eryngii* were prepared to investigate its effect on the viscoelastic properties of dough. SPPE addition levels were designed according to PEP content. The viscoelasticity of dough with SPPE addition is presented in Figure 4. The results showed that G′ and G″ of doughs first decreased at the 1.0% SPPE level and then increased with increasing SPPE level (Figure 4a,b), while tan δ of doughs first increased at the 1.0% and 2.0% SPPE levels and then increased as the SPPE level increased (Figure 4c). The variational tendency of G′, G″, and tan δ had no correlations (*ρ* = −0.051, *p > *0.05; *ρ* = 0.177, *p > *0.05; *ρ* = 0.302, *p > *0.05) with that of SPPE addition levels (Table 3). Furthermore, G′ and G″ of doughs with SPPE addition were all lower than those of control dough, while the tan δ values of doughs with SPPE addition were all higher than that of control dough. These results revealed that SPPE addition had weakened the gluten network structure of dough, and the weakening effect was more significant than that of the equivalent addition level of PEP. This might be attributed to the easy formation of hydrogen bonds between SPPE and water molecules (Lerbret et al., 2005), which destroys the formation of gluten network in the dough (Peng et al., 2017). Blanco Canalis et al. (2017) reported an analogous result suggesting that inulin can partially enter the aqueous phase of dough, which lead to an increase of volume and reduce the firmer consistency of dough. In addition, SPPE has good water solubility and swells sufficiently in dough, which results in more viscous properties of dough but less elastic and firmer consistency. Furthermore, compared to SPPE, there are a large number of other components in PEP, such as IDF (32.72%), protein (16.39%), crude fat (5.13%), and other small‐molecular substances, which might react with wheat‐flour starch and proteins and thereby also affect the viscoelastic properties of dough. These factors probably resulted in the more significant effects of SPPE on G′, G″, and tan δ compared to those of equivalent levels of PEP. Table 3 also showed that the viscoelastic properties of SPPE dough had no correlations (*ρ* = 0.471, *p > *0.05; *ρ* = 0.630, *p > *0.05; *ρ* = 0.416, *p > *0.05) with that of PEP dough.

### 3.5 Microstructure of composite dough

**Figure 5:**
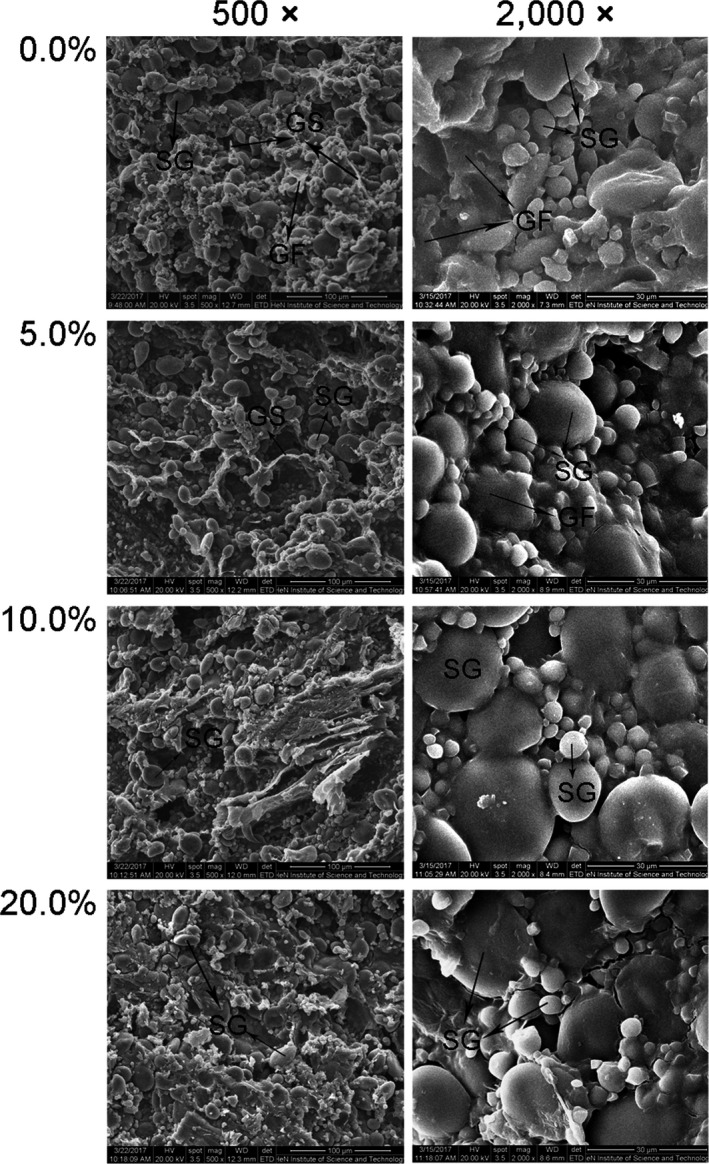
Scanning electron micrographs of composite dough supplemented with PEP at various levels. GF, gluten film; GS, gluten strand; SG, starch granule

The microstructures of the doughs with different addition levels of PEP were observed by SEM, as shown in Figure 5. In the micrographs of the control sample (wheat‐flour dough), the starch granules (SG) were tightly embedded in the integrated and continuous gluten network composed of gluten strand (GS) and gluten film (GF). While with the increase of PEP level, the integrity and continuity of the gluten network became increasingly weaker. At a PEP level of 5%, the gluten strand became thinner than that of the control. When the PEP level reached 10%, few gluten strands could be found, and the gluten film became exiguous. When the PEP level was 20%, some fiber‐like structures could be observed, and the number of starch granules decreased accordingly; meanwhile, the gluten network was difficult to find.

**Figure 6:**
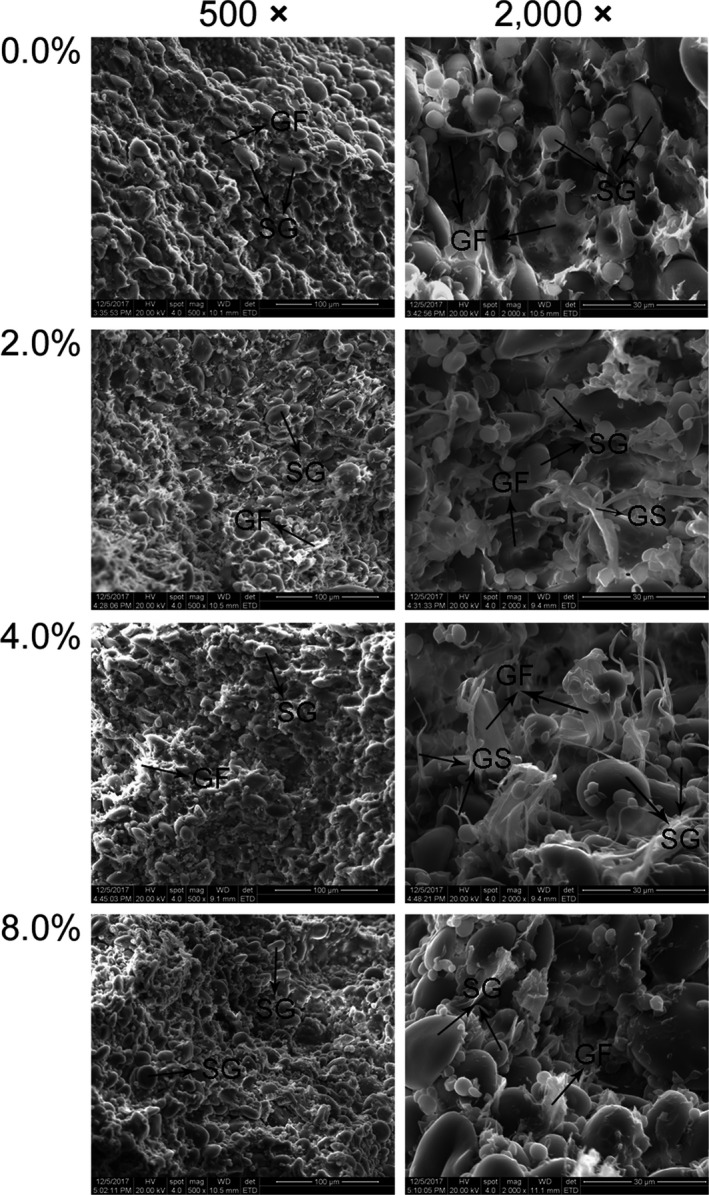
Scanning electron micrographs of composite dough supplemented with SPPE at various levels. GF, gluten film; GS, gluten strand; SG, starch granule

To further investigate the effect of polysaccharides in *P. eryngii* on the structure of the dough, part of the wheat flour was replaced with SPPE to make dough. The microstructures of the doughs with different levels of SPPE had analogous results to those with PEP (Figure 6). The addition of PEP and SPPE both disrupted the continuity of the gluten network and enlarged the hollows and voids in the dough. Although some portions of the GF could be observed in the dough with SPPE addition, they could not completely wrap the SG, which appeared less embedded in the gluten network than those in the control sample. The SEM results showed that PEP or SPPE addition destroyed the integrity and continuity of the gluten network and thus resulted in a dough with weak extensibility, which was consistent with the rheological results as previously mentioned.

## 4 CONCLUSION

This study demonstrated that different addition levels of PEP and SPPE affected the rheological properties and microstructures of dough systems. Farinographic assay results suggested that PEP could increase the water absorption and decrease the development time and stability of dough, while extensographic assays indicated that it was capable of providing weak extensographic characteristics and harder dough. The dynamic oscillatory tests revealed that the PEP addition roughly increased G′ and G″ but decreased tan δ in the entire frequency range, while the addition of SPPE had results inconsistent with those of PEP addition. The microstructure of dough samples was observed by SEM, which showed that the PEP and SPPE addition both decreased the integrity and continuity of gluten network in the dough. Overall, adding PEP produced a significant effect on the quality of dough, and relatively low addition levels of 2.5%–5.0% are recommended. This research could provide a foundation for the application of PEP in wheat‐flour foods. Further research will be conducted to investigate the influence of PEP addition on dough‐based foods like steamed bread, noodles, and biscuits, and products with high nutritional and edible qualities will be developed.

## CONFLICT OF INTEREST

The authors declare that they do not have any conflict of interest.

## ETHICAL STATEMENT

This study does not involve any human or animal testing.

## References

[fsn31054-bib-0001] AACC International (2000). Methods 44–15, 08–01, 46–11, 30–10, 80–68, 54–21, 38–12, 44–19. Approved methods of the American Association of Cereal Chemists, 10th ed St. Paul, MN: The American Association of Cereal Chemist Inc.

[fsn31054-bib-0002] Ahmed, J. , Almusallam, A. S. , Al‐Salman, F. , Abdulrahman, M. H. , & Al‐Salem, E. (2013). Rheological properties of water insoluble date fiber incorporated wheat flour dough. LWT – Food Science and Technology, 51, 409–416. https://doi.org/10.1016j.lwt.2012.11.018

[fsn31054-bib-0003] AOAC Official Methods 991.43 (2000). Total, soluble and insoluble dietary fibre in foods, Enzymatic‐Gravimetric method, Final action 1994 In Indyk H., & Konings E. (Eds.), Official methods of analysis of AOAC international, 17th ed.

[fsn31054-bib-0004] Blanco Canalis, M. S. , Steffolani, M. E. , León, A. E. , & Ribotta, P. D. (2017). Effect of different fibers on dough properties and biscuit quality. Journal of the Science of Food & Agriculture, 97, 1607–1615. 10.1002/jsfa.7909 27418199

[fsn31054-bib-0005] Chang, H.‐Y. , Hu, Y.‐W. , Yue, C.‐S. , Wen, Y.‐W. , Yeh, W.‐T. , Hsu, L.‐S. , … Pan, W.‐H. (2006). Effect of potassium‐enriched salt on cardiovascular mortality and medical expenses of elderly men. American Journal of Clinical Nutrition, 83, 1289–1296. 10.1093/ajcn/83.6.1289 16762939

[fsn31054-bib-0006] Chen, J. J. , Mao, D. , Yong, Y. Y. , Li, J. L. , Wei, H. , & Lu, L. (2012). Hepatoprotective and hypolipidemic effects of water‐soluble polysaccharidic extract of *Pleurotus eryngii* . Food Chemistry, 130, 687–694. 10.1016/j.foodchem.2011.07.110

[fsn31054-bib-0007] Correa, M. J. , Añón, M. C. , Pérez, G. T. , & Ferrero, C. (2010). Effect of modified celluloses on dough rheology and microstructure. Food Research International, 43, 780–787. 10.1016/j.foodres.2009.11.016

[fsn31054-bib-0008] Du, Z. , Chen, F. , Liu, K. , Lai, S. , Zhang, L. , Bu, G. , … Liu, S. (2016). Effects of extruded soy protein on the quality of Chinese steamed bread. Journal of Chemistry, 2016, 2113–8. 10.1155/2016/3691523

[fsn31054-bib-0009] Dubois, M. , Gilles, K. A. , Hamilton, J. K. , Rebers, P. A. , & Smith, F. (1956). Colorimetric method for determination of sugars and related substances. Analytical Chemistry, 28, 350–356. 10.1021/ac60111a017

[fsn31054-bib-0010] Edwards, N. M. , Dexter, J. E. , & Scanlon, M. G. (2002). Starch participation in durum dough linear viscoelastic properties. Cereal Chemistry, 79, 850–856. 10.1094/CCHEM.2002.79.6.850

[fsn31054-bib-0011] Fu, J. T. , Chang, Y. H. , & Shiau, S. Y. (2015). Rheological, antioxidative and sensory properties of dough and mantou (steamed bread) enriched with lemon fiber. LWT – Food Science and Technology, 61, 56–62. 10.1016/j.lwt.2014.11.034

[fsn31054-bib-0012] Gökşen, G. , & Ekiz, H. İ. (2016). Effect of *Prunus mahaleb* seed powder on dough rheology and bread quality. Journal of Food Quality, 39, 436–444. 10.1111/jfq.12220

[fsn31054-bib-0013] Goldstein, A. , Ashrafi, L. , & Seetharaman, K. (2010). Effects of cellulosic fibre on physical and rheological properties of starch, gluten and wheat flour. International Journal of Food Science and Technology, 45, 1641–1646. 10.1111/j.1365-2621.2010.02323.x

[fsn31054-bib-0014] Jeong, Y. T. , Jeong, S. C. , Gu, Y. A. , Islam, R. , & Song, C. H. (2010). Antitumor and immunomodulating activities of endo‐biopolymers obtained from a submerged culture of *Pleurotus eryngii* . Food Science & Biotechnology, 19, 399–404. 10.1007/s10068-010-0056-4

[fsn31054-bib-0015] Lerbret, A. , Bordat, P. , Affouard, F. , Guinet, Y. , Hédoux, A. , Paccou, L. , … Descamps, M. (2005). Influence of homologous disaccharides on the hydrogen‐bond network of water: Complementary Raman scattering experiments and molecular dynamics simulations. Carbohydrate Research, 340, 881–887. 10.1016/j.carres.2005.01.036 15780254

[fsn31054-bib-0016] Li, M. , Zhu, K. , Guo, X. , Brijs, K. , & Zhou, H. (2014). Natural additives in wheat‐based pasta and noodle products: Opportunities for enhanced nutritional and functional properties. Comprehensive Reviews in Food Science & Food Safety, 13, 347–357. 10.1111/1541-4337.12066 33412715

[fsn31054-bib-0017] Liu, X. , Zhou, B. O. , Lin, R. , Jia, L. E. , Deng, P. , Fan, K. , … Zhang, J. (2010). Extraction and antioxidant activities of intracellular polysaccharide from *Pleurotus* sp. mycelium. International Journal of Biological Macromolecules, 47, 116–119. 10.1016/j.ijbiomac.2010.05.012 20580645

[fsn31054-bib-0018] Lu, X. , Brennan, M. A. , Serventi, L. , Mason, S. , & Brennan, C. S. (2016). How the inclusion of mushroom powder can affect the physicochemical characteristics of pasta. International Journal of Food Science & Technology, 51, 2433–2439. 10.1111/ijfs.13246

[fsn31054-bib-0019] McCann, T. H. , Le Gall, M. L. , & Li, D. (2016). Extensional dough rheology – Impact of flour composition and extension speed. Journal of Cereal Science, 69, 228–237. 10.1016/j.jcs.2016.03.012

[fsn31054-bib-0020] Mirsaeedghazi, H. , Emam‐Djomeh, Z. , & Mousavi, S. M. A. (2008). Rheometric measurement of dough rheological characteristics and factors affecting it. International Journal of Agriculture and Biology, 10, 112–119.

[fsn31054-bib-0021] Morris, C. , & Morris, G. A. (2012). The effect of inulin and fructo‐oligosaccharide supplementation on the textural, rheological and sensory properties of bread and their role in weight management: A review. Food Chemistry, 133, 237–248. 10.1016/j.foodchem.2012.01.027 25683391

[fsn31054-bib-0022] Oliver, W. J. , Cohen, E. L. , & Neel, J. V. (1975). Blood pressure, sodium intake, and sodium related hormones in the Yanomamo Indians, a "no‐salt" culture. Circulation, 52, 146–151. 10.1161/01.CIR.52.1.146 1132118

[fsn31054-bib-0023] Peng, B. , Li, Y. , Ding, S. , & Yang, J. (2017). Characterization of textural, rheological, thermal, microstructural, and water mobility in wheat flour dough and bread affected by trehalose. Food Chemistry, 233, 369–377. 10.1016/j.foodchem.2017.04.108 28530586

[fsn31054-bib-0024] Petitot, M. , Boyer, L. , Minier, C. , & Micard, V. (2010). Fortification of pasta with split pea and faba bean flours: Pasta processing and quality evaluation. Food Research International, 43, 634–641. 10.1016/j.foodres.2009.07.020

[fsn31054-bib-0025] Ploysangam, A. , Falciglia, G. A. , & Brehm, B. J. (1997). Effect of marginal zinc deficiency on human growth and development. Journal of Tropical Pediatrics, 43, 192–198. 10.1093/tropej/43.4.192-a 9283119

[fsn31054-bib-0026] Ramli, S. , Alkarkhi, A. F. , Shin, Y. Y. , Min‐Tze, L. , & Easa, A. M. (2009). Effect of banana pulp and peel flour on physicochemical properties and in vitro starch digestibility of yellow alkaline noodles. International Journal of Food Sciences & Nutrition, 60, 326–340. 10.1080/09637480903183503 19757248

[fsn31054-bib-0027] Ren, D. , Wang, N. , Guo, J. , Yuan, L. , & Yang, X. (2016). Chemical characterization of *Pleurotus eryngii* polysaccharide and its tumor‐inhibitory effects against human hepatoblastoma HepG‐2 cells. Carbohydrate Polymers, 138, 123–133. 10.1016/j.carbpol.2015.11.051 26794745

[fsn31054-bib-0028] Ribotta, P. D. , Ausar, S. F. , Beltramo, D. M. , & León, A. E. (2005). Interactions of hydrocolloids and sonicated‐gluten proteins. Food Hydrocolloids, 19, 93–99. 10.1016/j.foodhyd.2004.04.018

[fsn31054-bib-0029] Rubel, I. A. , Pérez, E. E. , Manrique, G. D. , & Genovese, D. B. (2015). Fibre enrichment of wheat bread with *Jerusalem artichoke* inulin: Effect on dough rheology and bread quality. Food Structure, 3, 21–29. 10.1016/j.foostr.2014.11.001

[fsn31054-bib-0030] Santiago, D. M. , Kawashima, Y. , Matsushita, K. , Noda, T. , Pelpolage, S. , Tsuboi, K. , … Yamauchi, H. (2016). Noodle qualities of fresh pasta supplemented with various amounts of purple sweet potato powder. Food Science & Technology Research, 22, 307–316. 10.3136/fstr.22.307

[fsn31054-bib-0031] Sheng, J. , Yu, F. , Xin, Z. , Zhao, L. , Zhu, X. , & Hu, Q. (2007). Preparation, identification and their antitumor activities in vitro of polysaccharides from *Chlorella pyrenoidosa* . Food Chemistry, 105, 533–539. 10.1016/j.foodchem.2007.04.018

[fsn31054-bib-0032] Shi, A. M. , Wang, L. J. , Li, D. , & Adhikari, B. (2013). Characterization of starch films containing starch nanoparticles. Part 2: Viscoelasticity and creep properties. Carbohydrate Polymers, 96, 602–610. 10.1016/j.carbpol.2012.10.064 23768606

[fsn31054-bib-0033] Sim, S. Y. , Aziah, A. A. N. , & Cheng, L. H. ( 2015). Quality and functionality of Chinese steamed bread and dough added with selected non‐starch polysaccharides. Journal of Food Science & Technology, 52, 303–310. 10.1007/s13197-013-0967-1

[fsn31054-bib-0034] Song, X. , Zhu, W. , Pei, Y. , Ai, Z. , & Chen, J. (2013). Effects of wheat bran with different colors on the qualities of dry noodles. Journal of Cereal Science, 58, 400–407. 10.1016/j.jcs.2013.08.005

[fsn31054-bib-0035] Sun, R. , Zhang, Z. , Hu, X. , Xing, Q. , & Zhuo, W. (2015). Effect of wheat germ flour addition on wheat flour, dough and Chinese steamed bread properties. Journal of Cereal Science, 64, 153–158. 10.1016/j.jcs.2015.04.011

[fsn31054-bib-0036] Sun, Y. , Hu, X. , & Li, W. (2017). Antioxidant, antitumor and immunostimulatory activities of the polypeptide from *Pleurotus eryngii* mycelium. International Journal of Biological Macromolecules, 97, 323–330. 10.1016/j.ijbiomac.2017.01.043 28093329

[fsn31054-bib-0037] Wandee, Y. , Uttapap, D. , Puncha‐Arnon, S. , Puttanlek, C. , Rungsardthong, V. , & Wetprasit, N. (2015). Enrichment of rice noodles with fibre‐rich fractions derived from cassava pulp and pomelo peel. International Journal of Food Science & Technology, 49, 2348–2355. 10.1111/ijfs.12554

[fsn31054-bib-0038] Xu, F. , Hu, H. , Liu, Q. , Dai, X. , & Zhang, H. (2017). Rheological and microstructural properties of wheat flour dough systems added with potato granules. International Journal of Food Properties, 20, S1145–S1157. 10.1080/10942912.2017.1337791.

[fsn31054-bib-0039] Yuan, B. , Zhao, L. , Rakariyatham, K. , Han, Y. , Gao, Z. , Muinde Kimatu, B. , … Xiao, H. (2017). Isolation of a novel bioactive protein from an edible mushroom *Pleurotus eryngii* and its anti‐inflammatory potential. Food & Function, 8, 2175–2183. 10.1039/C7FO00244K 28524200

[fsn31054-bib-0040] Yuan, B. , Zhao, L. , Yang, W. , McClements, D. J. , & Hu, Q. (2017). Enrichment of bread with nutraceutical‐rich mushrooms impact of *Auricularia auricula* (mushroom) flour upon quality attributes of wheat dough and bread. Journal of Food Science, 82, 2041–2050. 10.1111/1750-3841.13812 28753727

[fsn31054-bib-0041] Zeng, X. Y. , Wang, M. F. , Zhu, B. K. , Liang, H. , Liao, T. G. , Wang, S. Y. , & Zhe, W. (2013). Simultaneous analysis of ay and amino acids in corn oligopeptides by HPLC‐fluorescence detector with OPA/FMOC‐Cl pre‐column derivatization. International Journal of Food Agriculture & Environment, 11, 86–90.

[fsn31054-bib-0042] Zhang, C. , Zhang, L. , Liu, H. , Zhang, J. , Hu, C. , & Jia, L. (2018). Antioxidation, anti‐hyperglycaemia and renoprotective effects of extracellular polysaccharides from *Pleurotus eryngii* SI‐04. International Journal of Biological Macromolecules, 111, 219–228. 10.1016/j.ijbiomac.2018.01.009 29309869

[fsn31054-bib-0043] Zhou, Y. B. , Wang, D. F. , Wan, X. C. , Zhang, L. , Du, X. F. , & Hu, W. S. (2009). Effect of tea polysaccharide addition on the properties of bread made from two flours. Journal of Food Processing and Preservation, 33, 798–813. 10.1111/j.1745-4549.2008.00312.x

[fsn31054-bib-0044] Zhu, F. , Sakulnak, R. , & Wang, S. (2016). Effect of black tea on antioxidant, textural, and sensory properties of Chinese steamed bread. Food Chemistry, 194, 1217–1223. 10.1016/j.foodchem.2015.08.110 26471674

